# Renal cell tumour characteristics in patients with the Birt-Hogg-Dubé cancer susceptibility syndrome: a retrospective, multicentre study

**DOI:** 10.1186/s13023-014-0163-z

**Published:** 2014-10-29

**Authors:** Patrick R Benusiglio, Sophie Giraud, Sophie Deveaux, Arnaud Méjean, Jean-Michel Correas, Dominique Joly, Marc-Olivier Timsit, Sophie Ferlicot, Virginie Verkarre, Caroline Abadie, Dominique Chauveau, Dominique Leroux, Marie-Françoise Avril, Jean-François Cordier, Stéphane Richard, on behalf of the French National Cancer Institute « Inherited predisposition to Kidney Cancer » network

**Affiliations:** Centre Expert National Cancers Rares PREDIR, Hôpital Bicêtre, AP-HP, Batiment Lasjaunias, 78 rue du Général Leclerc, 94275 Le Kremlin Bicêtre, France; Consultation d’Oncogénétique, Département de Médecine Oncologique, Gustave Roussy Cancer Campus, 114 rue Edouard Vaillant, 94805 Villejuif, France; Génétique Moléculaire et Clinique, Hôpital Edouard Herriot, 5 place d’Arsonval,, 69437 Lyon, France; Service d’Urologie, Hôpital européen Georges-Pompidou, AP-HP, 20 rue Leblanc, 75908 Paris, France; Université Paris-Descartes, 12 Rue de l’École de Médecine, 75006 Paris, France; Service de Radiologie Adulte, Hôpital Necker-Enfants Malades, AP-HP, 149 rue de Sèvres, 75743 Paris, France; Service de Néphrologie, Hôpital Necker-Enfants Malades, AP-HP, 149 rue de Sèvres, 75743 Paris, France; Service d’Anatomie Pathologique, Hôpital Bicêtre, AP-HP, 78 rue du Général Leclerc, 94275 Le Kremlin Bicêtre, France; Service d’Anatomie Pathologique, Hôpital Necker-Enfants Malades, AP-HP, 149 rue de Sèvres, 75743 Paris, France; Service de Génétique Médicale, CHU Rennes, 16 Boulevard de Bulgarie, 35203 Rennes, France; Département de Néphrologie et Transplantation d’Organes, INSERM UMR 1048, Hôpital de Rangueil, 1 avenue Poulhès, 31059 Toulouse, France; Département d’Hématologie, Oncogénétique et Immunologie, CHU de Grenoble site Nord - Institut de biologie et de pathologie, Boulevard de la Chantourne, 38700 La Tronche, France; Service de Dermatologie, Hôpital Cochin, AP-HP, 27 rue du Faubourg Saint-Jacques, 75679 Paris, France; Centre de Référence des Maladies Pulmonaires Rares, Service de Pneumologie, Hôpital Louis Pradel, 28 avenue du Doyen Lépine, 69677 Lyon, France; Génétique Oncologique EPHE, INSERM U743, Institut de cancérologie Gustave Roussy, 94800 Villejuif and Faculté de Médecine Paris-Sud, 94276 Le Kremlin-Bicêtre, France

**Keywords:** Renal cell carcinoma, Neoplastic syndromes, Hereditary, Birt-Hogg-Dubé, Genetic predisposition to disease, Folliculin

## Abstract

**Background:**

The Birt-Hogg-Dubé syndrome is a rare cancer susceptibility syndrome characterised by renal tumours, lung cysts and pneumothoraces, and fibrofolliculomas. It is caused by dominantly inherited mutations in *FLCN*. Our objective was to report renal tumour characteristics in a large series of patients with the Birt-Hogg-Dubé syndrome.

**Methods:**

We studied French Birt-Hogg-Dubé patients with a history of renal tumour.

**Results:**

We included 33 patients with 21 distinct germline *FLCN* mutations. Median age at diagnosis of first renal tumour was 46, and age varied from 20 to 83. Twenty cases had one renal tumour, the remainder had two or more tumours. Most cases (23/33, 70%) had oncocytoma or renal cell carcinoma of the chromophobe or hybrid chromophobe-oncocytoma type, three had clear cell carcinoma (9%), and the other seven had carcinoma of papillary, undifferentiated or undetermined histology. Four cases had metastatic disease, although none died of it.

**Conclusions:**

Age at renal tumour diagnosis was highly variable, highlighting the need for regular surveillance from young adulthood to old age. Most cases had tumour types classically associated with Birt-Hogg-Dubé, i.e. oncocytoma or renal cell carcinoma of the chromophobe or hybrid type. Nevertheless, 9% had clear cell renal cell carcinoma. Geneticists, urologists and oncologists should therefore be alert to the possibility of Birt-Hogg-Dubé in patients with renal cell carcinoma of clear cell histology, especially if there are associated manifestations. Finally, the behaviour of metastatic carcinoma seemed more indolent than in sporadic renal cancers.

## Introduction

The Birt-Hogg-Dubé syndrome (BHD) is a rare cancer susceptibility syndrome characterised mainly by renal cell tumours, lung cysts and pneumothoraces, and skin papules named fibrofolliculomas [1]. It is caused by germline mutations in the *FLCN* gene, which are inherited in a dominant fashion [1]. *FLCN* codes for folliculin, a protein that operates in the mTOR pathway and leads, when inactivated, to increased mitochondrial oxidative metabolism [2,3]. Phenotype is variable among patients with BHD. Indeed, renal cell tumours affects up to 34% of mutation carriers, and consists predominantly of chromophobe renal cell carcinomas (RCC), benign oncocytomas, and RCC with hybrid chromophobe – oncocytoma histology [4-7]. Twenty-four to 37% of BHD cases present with at least one episode of pneumothorax in their lifetime, and 79% to 84% have skin fibrofolliculomas [4,5,8].

We describe herein renal cell tumour characteristics in 33 BHD patients, among them four cases with metastatic, albeit indolent disease.

## Patients and methods

In France, patients are referred to accredited cancer genetics clinics throughout the country when genetic susceptibility to renal cell tumours is suspected. Not all patients present with a personal or family history of renal cell tumour. Indeed, some have non-renal manifestations suggestive of a syndrome in which renal cell tumour risk is increased, and others are healthy relatives of individuals with an established genetic predisposition. The genes most commonly analysed in hereditary RCC are *VHL* (associated with von Hippel-Lindau disease)*, FH* (Hereditary Leiomyomatosis with RCC)*, FLCN* (BHD), *SDHB* (PGL4 syndrome), and *MET* (Hereditary Papillary RCC) [9].

On November 15, 2013, we explored two overlapping sources, the PREDIR National Hereditary Kidney Cancer Centre database located at Hôpital Bicêtre near Paris, and the Molecular Genetics Laboratory at the Edouard Herriot University Hospital in Lyon. The PREDIR centre strives to collect clinical and molecular data on all French carriers of a mutation predisposing to renal cell tumours, whereas the Lyon laboratory is the main provider of *FLCN* germline analysis in France. We retrieved clinical data for all patients with a diagnosis of molecularly-proven BHD, and selected those with a history of renal cell tumour at the time of genetic testing. All patients had signed an informed consent form before genetic testing, as legally required. Cases reported in two previous papers are included in this study [10,11]. Follow-up data for included patients are up-to-date as of 29 September 2014, the day the final version of this manuscript was submitted.

Mutations were identified using both Sanger sequencing of exons and their flanking regions and multiplex ligation probe-dependent amplification (MLPA) on genomic DNA extracted from blood. Detailed protocols are available on request. Mutations were considered pathogenic if they had been reported and reliably identified as such in the *FLCN* mutation database (http://grenada.lumc.nl/LOVD2/shared1), or for never-reported mutations, if they led to a truncated protein.

## Results

**Figure 1:**
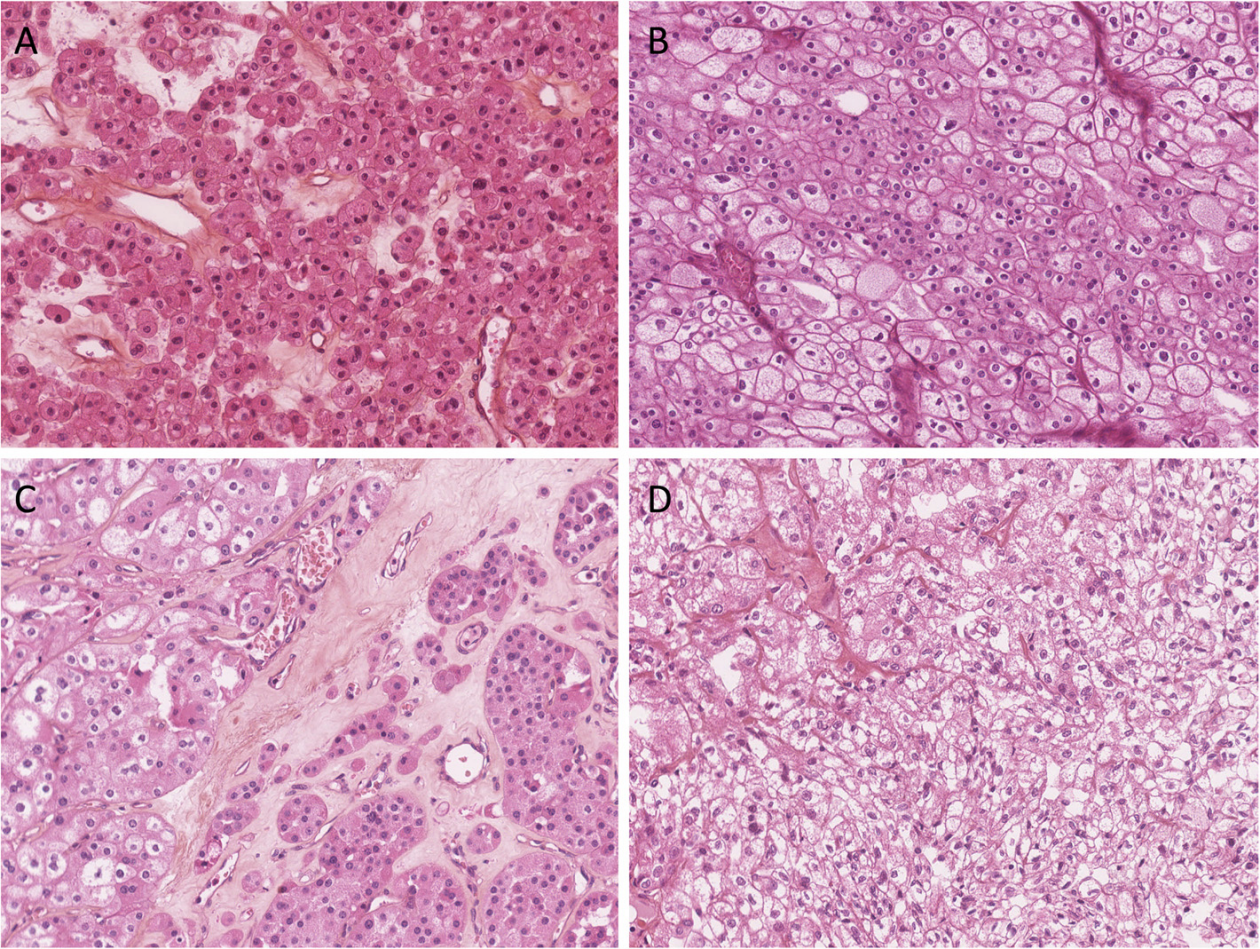
**Main types of renal tumours seen in our patients with BHD.** Oncocytoma **(A)**, chromophobe carcinoma **(B)**, hybrid chromophobe-oncytoma carcinoma **(C)**, and clear cell carcinoma **(D)**.

We ascertained a total of 124 BHD patients from 72 distinct families. Thirty-three had a history of renal cell tumour (Tables 1 and 2). Twenty-one were male, 12 female. Median age at diagnosis of first tumour was 46 (age range 20–83). Twenty cases had one tumour, four had two tumours, and nine had multifocal (three or more) tumours. Most cases (23/33, 70%) had oncocytoma or RCC of the chromophobe or hybrid chromophobe-oncocytoma type, three had clear cell RCC (9%), one had papillary RCC with interspersed eosinophilic cells, and one had undifferentiated RCC. Histopathology was unavailable for five patients, most often because their tumours were radiologically characteristic but too small to justify surgery. The most common histological types are illustrated in Figure 1.

**Table 1:** **Birt-Hogg-Dubé patients with renal tumours**

**Patient**	**Gender**	**Mutation**	**1st renal tumour, age**	**Number of renal tumours**	**Histology**	**Other BHD manifestations**	**Duration of F-U (months)**	**Latest F-U data**
11525	M	c.1285dup, p.His429Profs*27	73	Multifocal	Ch, H	FF	30	05/2014
11528	M	c.1285dup, p.His429Profs*27	83	2	N/A	FF, PNO	103	11/2013
11545	M	c.1285dup, p.His429Profs*27	39	1	N/A	Lung cysts	65	05/2011
13851	M	c.1285dup, p.His429Profs*27	35	1 (Mt)	Und	FF	225	03/2003 (D)
14381	M	c.755dup, p.Cys253Valfs*39	62	1	CC	FF	150	05/2009
14840	M	c.1285dup, p.His429Profs*27	20	1	N/A	FF, PNO	672	02/2011
15842	F	c.828del, p.Ala277Leufs*16	38	1	Ch	-	176	02/2014
19276	M	c.1062 + 2 T > G	56	2	Ch, H	FF	96	05/2011
19398	F	c.663dup, p.Met222Aspfs*26	42	Multifocal (Mt)	H	PNO	115	09/2014
20471	F	c.1285dup, p.His429Profs*27	34	Multifocal	Ch, O	FF, PNO	248	01/2014
20472	M	c.67G > T, p.Glu23*	43	1	Ch	PNO	132	03/2014
21310	F	c.1198G > A, p.Val400Ile	46	1(Mt)	Ch	-	99	07/2014
22973	M	c.1523A > G, p.Lys508Arg	56	1 (Mt)	Ch	FF, PNO	96	03/2014
25891	F	c.318C > G, p.Tyr106*	25	1	P	Lung cysts	96	03/2014
26016	M	c.1300G > A, p.Glu434Lys	48	Multifocal	Ch, H	FF	16	05/2011
27674	M	c.616A > T, p.Lys206*	56	1	Ch	PNO	93	05/2013
28190	M	c.1367_1398del, p.Asp456Glyfs*19	53	1	O	Lung cysts	16	09/2012
28535	F	c.1063-2A > G	51	1	Ch	FF, lung cysts	72	04/2014
28537	F	c.1300G > A, p.Glu434Lys	47	2	H	PNO	20	02/2014
28590	M	c.1285dup, p.His429Profs*27	47	1	Ch	FF, lung cysts	13	05/2013
31122	F	c.1285dup, p.His429Profs*27	41	Multifocal	CC	Lung cysts, PNO	264	06/2005
36616	M	c.1285del, p.His429Thrfs*39	40	1	N/A	FF	312	01/2008
37644	M	c.958dup, p.Arg320Profs*70	41	2	O	FF, PNO	6	07/2008
37673	M	c.610_611delinsTA, p.Ala204*	44	Multifocal	Ch	-	9	07/2008
40154	F	c.1528_1530del, p.Glu510del	30	Multifocal	O	-	N/A	02/2009
40893	M	c.323G > T, p.Ser108Ile	51	1	CC	FF, PNO	45	03/2013
42207	F	c.1300G > A, p.Glu434Lys	54	1	Ch	FF, PNO	74	01/2014
42337	M	c.1300G > A, p.Glu434Lys	50	1	H	FF, lung cysts, PNO	60	02/2014
47950	M	c.1458del, p.Ile486Metfs*5	44	Multifocal	O	FF	24	02/2014
47639	F	c.57_58del, p.Phe20Leufs*16	44	1	H	PNO	43	05/2014
48746	M	c.1318del, p.Glu440Argfs*28	54	1	Ch	FF, lung cysts	35	01/2014
48781	F	c.1285dup, p.His429Profs*27	55	1	N/A	FF, PNO	80	02/2013
50335	M	c.958dup, p.Arg320Profs*70	39	Multifocal	O	PNO	24	06/2014

All patients were alive at last follow-up, unless specified otherwise. CC, clear cell renal cell carcinoma. Ch, chromophobe renal cell carcinoma. D, deceased. FF, fibrofolliculomas, F-U, follow-up. H, hybrid chromophobe- oncocytoma renal cell carcinoma. Mt, metastatic disease. N/A, not available. O, oncocytoma. P, papillary renal cell carcinoma, type undetermined. PNO, pneumothorax. Und, undifferentiated.

**Table 2:** **Summary of clinical and histopathological characteristics of renal tumours among 33 patients with Birt-Hogg-Dubé**

	**Characteristics**	**Number of patients**
*Gender*	Male	21
	Female	12
*Tumour number*	1	20
	2	4
	Multifocal	9
*Tumour histology*	Oncocytoma/Chromophobe/Hybrid chromophobe- oncocytoma	23
	Clear cell	3
	Papillary (type undetermined)	1
	Undifferentiated	1
	N/A	5
*Extra-renal manifestations* ^§^	Lung cysts/pneumothorax	22
	Fibrofolliculomas	19
	None	4

^§^twelve patients had both pulmonary and dermatological manifestations. RCC, renal call carcinoma.

In addition to renal cell tumours, 29/33 cases also had documented extra-renal BHD manifestations. Twenty-two (67%) had lung cysts and/or pneumothoraces, and 19 (58%) had fibrofolliculomas (twelve cases had both pulmonary and dermatological manifestations).

There were 21 distinct mutations: frameshift (n = 10), missense (n = 4), nonsense (n = 4), splice site (n = 2) and in frame deletion (n = 1).

Among those 33 patients, four had metastatic RCC, either at the time of diagnosis or subsequently. All presented with symptoms that led to the diagnosis of RCC. Case 13851 had RCC age 35, he had undergone successful nephrectomy at the time. A solitary lung lesion was found incidentally age 58 on a CT scan that was prescribed after an episode of malignant hypertension. It was surgically removed and pathological examination showed it was a metastasis from a poorly differentiated RCC. The patient died of ischemic stroke four years after surgical resection of the metastasis, and there was no evidence of cancer relapse at the time. Case 19398 was diagnosed age 42 with bilateral multifocal hybrid chromophobe- oncocytoma RCC and liver metastases. Biopsy of a left renal tumour led to the pathological diagnosis. No further surgical procedure was carried out. For the first three years, she was under systemic therapy with sunitinib, everolimus and then temsirolimus, and disease stability was achieved. All treatments were then stopped. Eight years after the initial diagnosis, she is clinically well and the liver metastases are radiologically stable. Case 21310 had left nephrectomy age 46 for chromophobe RCC, and subsequently presented with liver and lung metastases. She is still alive five years after the metastases were first observed, even though slow radiological and clinical progression has justified treatment with sunitinib, axitinib, everolimus, bevacizumab and pazopanib. Finally, case 22973 had right nephrectomy age 56 for a chromophobe RCC. He received adjuvant radiotherapy for multiple, metastatic retroperitoneal lymph-nodes that could not be removed surgically. Seven years later, there are no radiological or clinical signs of disease progression.

## Discussion

Our series of 33 cases is one of the largest ever published [4-8]. This study confirms that cases can have either unifocal disease, or multiple tumours, and that most renal cell tumours are oncocytomas or RCC of chromophobe or hybrid chromophobe-oncocytoma histology [4-6]. Other histologies are however associated with the syndrome, clear cell in particular. Indeed, among our cases, 3 (9%) had clear cell carcinomas. Given that they are not typical in BHD, all clear cell RCC were reviewed by one of our expert pathologists, and confirmation was obtained. This proportion is similar to what has been reported in the past, and clinicians should therefore remain alert to the possibility of BHD in patients with clear cell RCC, especially if there are associated manifestations [6].

The age at renal cell tumour diagnosis varied between 20 and 83, with a median age of 46. Of special interest was the fact that two patients presented with a renal cell tumour in their twenties, highlighting the need for regular surveillance from an early age [12]. This surveillance should be continued with no upper age limit and as long as the patients are in good condition, as one patient with two tumours in his eighties was successfully treated by percutaneous radiofrequency ablation.

These 33 cases with renal cell tumours represent 27% of all ascertained BHD patients (33/124), and 34% of those for which we have evidence of renal imaging (33/98). This observation is remarkably consistent with two large studies from the United States National Cancer Institute that reported overall tumour frequencies of 29% and 34% in BHD patients evaluated by CT scan [4,5]. We expect the proportion in our study to be representative of the true renal cell tumour risk associated with the syndrome, as French index cases are offered *FLCN* genetic analysis because they present with all types of clinical manifestations suggestive of BHD.

An exhaustive description of extrarenal manifestations is beyond the scope of this paper. Nevertheless, 67% and 58% of our cases also had pulmonary and dermatological manifestations respectively, in addition to RCC. These figures represent the minimal prevalence as pulmonary assessment by CT scan and examination by a dermatologist knowledgeable in the syndrome was not systematic.

In our series, four patients had metastatic, albeit indolent RCC. Comprehensive family data was collected for all patients in this series, and we have no knowledge of other relatives (deceased or alive) with metastatic RCC who were not tested for *FLCN* mutations, and who were therefore not included in this study. Very few cases of metastatic RCC associated with BHD have been described. Pavlovich *et al.* and Houweling *et al.* reported respectively two and five such cases; all died rapidly despite multidisciplinary management that included surgery, radiotherapy, immunotherapy and chemotherapy, and the good prognosis associated with metastatic disease in our study contrasts with these earlier reports [8,13]. Three of the four patients reported here are still alive more than five years after the metastases were diagnosed, while the fourth died of unrelated causes four years after a lung metastasis had been removed. Whether these observations reflect a specific behaviour only seen in BHD requires further investigation. Another hypothesis is that metastatic RCC in BHD patients is more sensitive to tyrosine kinase and mTOR inhibitors such as sunitinib and everolimus (two recently-developed drugs that are now routinely used in the treatment of metastatic RCC), as suggested for example by the mTOR signalling pathway activation seen in kidney tumours associated with the syndrome, but that would not account for the good prognosis of cases 13851 and 22973 who received no systemic treatment [14]. As shown in Table 1, most patients in our study are followed up medically, with relevant information being collected by the team in charge on a regular basis. There is however a minority from which we have not heard in the last few years, and we cannot rule out with certainty the possibility of aggressive metastatic disease in these patients.

## Conclusions

The eponym syndrome was first described over 35 years ago by Birt, Hogg and Dubé [15]. However, the *FLCN* gene was only identified in 2002 [16]. The recent molecular characterisation of the syndrome, added to the fact that like most genetic diseases BHD is rare in the general population, means there are only limited data on the topic in the literature. As a result, this large and multicentric study will improve knowledge of the syndrome among geneticists, urologists and oncologists involved in the assessment and management of patients with a suspected or established genetic predisposition to renal cell tumours. It is of utmost importance to identify individuals with a high probability of developing renal cell tumours, so that they, and their at-risk relatives can benefit from personalised surveillance and management.
